# Supplementary material for: The Impact of a Post-Prescription Review and Feedback Antimicrobial Stewardship Program in Lebanon
Source: Antibiotics (Basel). 2022 May 11;11(5):642. doi: 10.3390/antibiotics11050642 (PMC9138162; doi:10.3390/antibiotics11050642)
Supplement: Supplementary file 1 [file antibiotics-11-00642-s001.zip › antibiotics-1647106-supplementary.pdf]

Supplementary Table S1. Physician survey responses: basic demographics.

Characteristic	Total (n = 20)
Gender	
Female	8
Male	12
Age, median	29.5<br>Range: 25–69
Specialty	
Medicine	14
Surgery	4
Intensive care unit	0
Other	2
Years since completion of highest education, median	6<br>Range: 1–40
Position at hospital	
Head of medical service	5
Head of surgical service	2
Medical consultant	3
Surgical consultant	1
Resident in medicine	7
Resident in surgery	1
Pharmacist	0
Other	1
Hospital department	
Medicine	15
Surgery	4
Intensive care unit	1
Years in current position, median	4
Years at current hospital, median	6.5

Supplementary Table S2. Physician survey responses on perceptions, knowledge, systemic failures and barriers, and attitudes towards a post-prescription review and feedback program (n = 20).

	Strongly Disagree	Disagree	Agree	Strongly Agree
**Perceptions and attitudes regarding antibiotics**				
Inappropriate antibiotic prescribing puts patients at risk	1	0	1	18
It is always better to over-prescribe than under-prescribe antibiotics	7	11	1	0
New antibiotics are being developed to solve the problem of resistance	2	4	10	3
In case of doubt, it is preferable to use a wide spectrum antibiotic to ensure the patient is cured of an infection	0	3	15	2
As a healthcare provider, I can contribute to appropriate use of antibiotics	0	0	7	13
Antimicrobial resistance is a problem in my daily practice	0	2	6	11
Antimicrobial resistance is a problem in the hospital where I work	0	0	11	8
Antimicrobial resistance is a problem in Lebanon	0	0	6	14
Antimicrobial resistance is a problem worldwide	0	0	7	13
**Knowledge and resources**				
I am aware of the antimicrobial resistance rates and patterns at the hospital where I work	1	5	8	6
I feel confident about my knowledge for prescribing antibiotics for my patients	0	6	9	5
I have received sufficient training in antibiotic prescribing through my workplace	1	6	10	3
I have received sufficient training in antibiotic prescribing through the Ministry of Health	12	7	1	0
I have received sufficient training in antibiotic prescribing through NGOs or other resources	12	7	1	0
I keep up with the most recent scientific literature on antibiotic resistance	0	7	7	5

I am aware of the Ministry of Health approved antibiotic treatment guidelines	4	9	5	2
**Systemic facilitators and barriers to antimicrobial stewardship**				
The hospital where I work has rapid and effective diagnostic techniques for diagnosis of infectious diseases	0	2	13	5
The hospital where I work provides sufficient guidelines for diagnosis and management of patients with infections	1	5	13	1
The hospital where I work has implemented rigorous infection control measures	0	2	12	6
In the hospital where I work, guidelines are appropriate for the setting	0	4	11	4
Pharmacists and medicine shops should not be allowed to sell antibiotics without a physician prescription	1	0	2	17
Healthcare providers should be compensated for prescribing antibiotics	13	2	1	1
The Ministry of Health should develop more stringent regulations regarding antibiotics	0	0	7	13
Healthcare providers need to do more to educate patients/consumers about antibiotic use and resistance	0	0	9	11
The Ministry of Health should develop campaigns to educate patients/consumers about antibiotic use and resistance	0	0	8	12
There is a need to increase education for physicians working outside of hospital settings (clinic) about antimicrobial resistance	0	0	8	12
There is a need to increase education for nurses and other healthcare providers about antimicrobial resistance	0	0	9	11
There is a need to increase education for nurses and other healthcare providers about antimicrobial resistance	0	0	7	13
There is a need to increase education for community pharmacists about antimicrobial resistance	0	0	5	15
**Post-prescription review and feedback (PPRF) program**				
The PPRF will increase antimicrobial stewardship in the ward where I work	0	0	10	9
The PPRF will provide me with new knowledge about appropriate antibiotic use	0	0	11	9
The PPRF will increase my confidence in use of antibiotics within my medical practice	0	0	12	8
The PPRF could be disruptive to my treatment of patients	4	13	2	1
Changing prescribed medications is costly for patients	2	7	11	0
Using the recommended changes could decrease risk of complications for my patients	0	0	11	9
In the long term, the PPRF could decrease antimicrobial resistance in my country	0	1	10	9

NGO: non-governmental organization.

Supplementary Table S3. Physician survey responses on supports and rewards for antimicrobial stewardship.

	Often	Sometimes	Never
I have discussed antibiotic treatment and resistance with hospital administrators	4	11	5
I have discussed antibiotic treatment and resistance with other health providers at the hospital where I work	12	8	0
I have discussed antibiotic treatment and resistance with other health providers outside of the hospital where I work	3	8	9
I have prescribed antibiotics because patients insist on it	2	4	14
I have prescribed antibiotics so that patients continue to trust me	1	2	16
I have prescribed antibiotics to improve my relationship with patients	1	1	18
I have had problems explaining to patients why they should not use antibiotics for certain conditions	7	11	2
I have felt that patients will use antibiotics whether I tell them to or not	5	11	4

Supplementary Table S4. Physician survey responses on useful means of increasing healthcare provider knowledge about antimicrobial resistance and treatment practices.

	Very Useful	Useful	Not Very Useful
Seminars or workshops	15	5	0
Summary written materials (e.g., reports)	12	8	0
Scientific articles	10	10	0
Videoconferencing	8	9	3
Internet sites	8	9	3

Mentoring	8	9	0
Short printed guidelines (e.g., pocket guide)	15	4	1
Poster with printed guideline	7	7	1
Webinars	6	10	4
